# Supplementary material for: A qualitative exploration of barriers to efficient and effective structured medication reviews in primary care: Findings from the DynAIRx study
Source: PLoS One. 2024 Aug 30;19(8):e0299770. doi: 10.1371/journal.pone.0299770 (PMC11364411; doi:10.1371/journal.pone.0299770)
Supplement: S2 Appendix — (DOCX) [file pone.0299770.s002.docx]

| **Theme: Medication Reviews in Practice** | |
| --- | --- |
| **Subtheme** | **Supporting quotes** |
| Limited availability of digital tools to assist in identifying and prioritising patients for a SMR | I haven’t got any digital tools to do structured medication reviews at the moment (Participant 3, Pharmacist FG2)  Are we prioritizing them? And I think it's difficult to really to build a search which would pinpoint exactly who you want to speak to you. You have to generate a search which kind of casts the net out wide and then you have to eyeball that list to see who then is a priority that slows things down…they still produce a vast list of patients really. And although they it is useful to have a that list that it's still difficult to identify within that list who should be prioritised… We need other tools now. Really. We we're crying out for things to identify these patients, to pinpoint them. Because we at the moment we just have searches really and then it's just clinical judgment. We need a bit more of a system which will you know filter through these patients pinpointing who are the most high risk ones. (Pharmacist 2, Interview)  If you’ve got 3000 care home residents, now, I don’t know which one to go to and, sort of, what I’ve often wanted to do, and, you know, we struggled a lot back in the day, was actually, you know, it would be very, it would be great if clinicians could actually just get a list of patients who we’re seeing next week. (Policy-maker, Interview)  Yeah our medicines team locally did some really good work around sort of which patients we should be focusing on and so it was yeah sort of frailty, polypharmacy and complex meds but as I say the IIF targets have slightly blown that out for England GPs because they’ve just got lots of people and some of them are just patients who happen to be on, sort of Naproxen and an NSAID and not much else and its really, it’s unfortunate because I think actually we are wasting resource you know limited resource. So, I think it is, you know focusing on, you know we had a template that we run when we are doing a med review and it would highlight the people who actually probably should be referred through for a structured medication review rather than just the GP proceeding you know sort of when they’re going through the meds. So that was quite a good filtering process. (Participant 4, GP FG1) |
| Organisational challenges and patient factors affecting patient engagement for a SMR | So I’ve moved from a practice which was fairly large and they had a really good, very efficient pharmacy team. So, lead pharmacist and then his you know his team members as well. And then I’ve moved to a practice now which is a very rural practice where I am doing medication reviews which means I feel like I’m a little bit out of depth because I’ve got used to the, of you know, all of that being done by a very efficient team who was looking at everything so they would look at you know the indications and the bloods, the and everything. So, this is one of the things that I’m currently trying to get used to. (Participant 2, GP FG1)  One of the difficulties we have is just getting people to come in, I work in a fairly deprived area and the only time we really get to see people is when they want some new medication. So, a lot of the medication reviews tend to be opportunistic medicine reviews that are done by the GP on the day which would definitely feed into what I would want from a system to help me later in the session. (Participant 1, GP FG1) |
| Time consuming “detective work” | Perhaps the bigger thing is just the density of data that we have on these patients. If they’ve got polypharmacy they’ve got polymorbidity too and they are the patients with the huge great files who you can never find any information on. So, you don’t know for example when their last medication review was. You don’t know when the last discussion was, you don’t know you know the different blood tests required for different medication. So that’s the problem is searching around for all that information and then checking the latest guidance for it I think. That’s probably the biggest thing for me which just adds up to time really. (Participant 1, GP FG1)  So when I’m playing pharmacy detective I, and I can’t get the information that I need, which is literally when something was started, stopped, the why out of the available, out of the access that I have in the computer systems which are really well linked and the patient and their relatives, my next step is a really beautiful conversation with a GP receptionist, which having then searched through and like I’m literally talking through what I know is in the computer system and they just need to find it and read it out to me so I can have my answer but knowing, if I know, if I can look, so I can see quite a lot in care. So, if I know when there has been a diagnosis or when I can see that a prescription was last issued for whatever it is I’m trying to work out I also get the first authorisation date for some systems, so I can direct the receptionist to like this time period, can you find me anything that mentions this in this timeframe. It would be way better if the receptionist was a computer and I didn’t have to wait for them to answer the phone. (Participant 2, Pharmacist FG1) |
| SMRs require multiple appointments | We require 3/4/5 appointments, sometimes with patients. They’re so complex. (Pharmacist 2, Interview)  Generally, like as a guide, I tend to try and allow 10 minutes per condition so if a patient has got like a lot more than 3 I will usually sort of tell them that we will just address some of things today and then we will have like a follow up appointment and address the others in a separate appointment. (Participant 5, Pharmacist FG2)  And in our care home, I think we, I’d have to go back and find, find the details, but I think we had contact on average about 2 to 3 times per patient. (Policy-maker, Interview) |
| Influence of healthcare context on delivering SMR | For frailer patients … the ones in the local care homes get done automatically, generally every six months or so, or whenever we make changes. I have to admit we also have some that we’ve given up on and those are usually, we have a local private hospital where some prescribing is interesting, to say the least … So we only prescribe what we are happy [with] but some of the ones they are getting privately are quite interesting and quite concerning. (Participant 2, GP FG2)  We have a pharmacy team of 5 or 6 pharmacists and some pharmacy techs that support them from it. Our network is 72,000 patients and they are 5 practices … I get a monthly report on where we are against the IIF targets … They are all done by the pharmacists. They organise their own clinics, they book patients in for reviews. (Participant 1, GP FG2)  A lot of ours are completed with our medicines management pharmacist … Some of them lead to subsequent structured medication reviews and they’ve been contracted by the PCN to complete a certain number, so they do the majority of them. I do quite a lot of them and we’ve got a couple of GP registrars who are being trained up in how to do SMRs for particular chronic diseases of particular cohorts of patients. (Participant 4, Pharmacist FG2) |
| Factors influencing deprescribing discussions | So along with what you said about deprescribing SSRIs, especially, you know they’ve been on them for 4-5 years and they are adamant they don’t want to reduce them or stop them or have any sort of conversation about it, yeah, they’re quite challenging I think. Also, I think sometimes you can’t quite work out what medications people have been on. (Participant 6, GP FG1)  I think the biggest fear that patients have when they come for medication reviews is I’m going to stop a medication that they want to keep taking. That by far, that’s the number one thing is you’re not going to stop my morphine are you, or something along those lines. I think that often stops them coming in for medication reviews generally. (Participant 1, GP FG1) |

| **Theme: Medication-related Challenges** | |
| --- | --- |
| **Subthemes** | **Supporting Quotes** |
| Poor communication & data sharing between primary and secondary care | We tend to put the secondary care prescribed drugs on to our EMIS records even if we are not prescribing them as a hospital issue just so that we’ve got that you know that sort of check when we are doing medication. But I agree, I don’t think the specialists have the same sort of knowledge of the interactions and we get to rely on our IT systems. So, it is definitely a grey area there. (Participant 5, GP FG1)  We also have the back up of those safety systems and that can often be a challenge where the specialist has said something and you end up having to navigate that choice with the existing medication and sometimes stopping it and then the patient gets very upset because the specialist has told them they need to be on it and that can sometimes be good, sometimes be less helpful. (Participant 1, GP FG1) |
| Difficulties managing mental health medication | Because I had a patient, I don’t know why they started it in the first place. So, I think that the psychiatric, you don’t know. I don’t know, I don’t feel that confident adjusting psychiatric meds and I don’t really understand the risk, its all about risk benefit. I don’t understand the risks support for someone who is being deprescribed off psychiatric meds. (Participant 2, Polypharmacy FG)  When it came to things like monitoring anti-psychotics, that would actually fall off the wagon. It was really difficult then to get the actual review again in a similar way to when there was polypharmacy, so when memantine was used and for instance and then it might have been also an antidepressant and they just stabilised the patient, they’ve gone through hell and back trying to get them stabilised and then they’ve discharged them and things could change. (Participant 1, Pharmacist FG1) |
| Challenges around anticholinergic medicines | For the anticholinergic burden so it was quite highly kind of programmed into us and it was on the template as well. In terms of, so from point of identifying it we all kind of drilled in quite frequently about that and from point of the actual speaking to the patients or trying to reduce that, sometimes it was quite difficult to stop one anticholinergic and not replace it with another one in all honesty. Saying that, so every time there was a reduction it felt like a big score has just been, “oh I’ve just really nailed it this time!”, and then like 3 weeks later I find I go end up with the same patient and I find that another anticholinergic has been prescribed. (Participant 1, Pharmacist FG1)  So, an automatic printout of all the interactions and the anticholinergic burden so that you can immediately then, 'cos that takes me quite a lot of time to work those two things out. And that, particularly for frail old people, the anticholinergic burden I think is more important, with burdens of 15 or 20 who are cognitively impaired with memory problems, falling over and just so that would really speed things up. (Participant 2, Polypharmacy FG) |
